# The clinical role of multimodality imaging in the detection of prostate cancer recurrence after radical prostatectomy and radiation therapy: past, present, and future

**DOI:** 10.3332/ecancer.2015.570

**Published:** 2015-09-04

**Authors:** Francesco Paparo, Michela Massollo, Ludovica Rollandi, Arnoldo Piccardo, Filippo Grillo Ruggieri, Gian Andrea Rollandi

**Affiliations:** 1 Radiology Unit, Department of Diagnostic Imaging, E O Galliera Hospital, Mura delle Cappuccine 14, 16128 Genoa, Italy; 2 Nuclear Medicine Unit, Department of Diagnostic Imaging, E O Galliera Hospital, Mura delle Cappuccine 14, 16128 Genoa, Italy; 3 Klinikum Augsburg Radiologie, Stelingstrasse 2, 86156 Augsburg, Germany; 4 Radiotherapy Unit, Department of Diagnostic Imaging, E O Galliera Hospital, Mura delle Cappuccine 14, 16128 Genoa, Italy

**Keywords:** prostate cancer, magnetic resonance imaging, choline, positron emission tomography, fusion imaging

## Abstract

Detection of the recurrence sites in prostate cancer (PCa) patients affected by biochemical recurrence after radical prostatectomy (RP) and radiation therapy (RT) is still a challenge for clinicians, nuclear medicine physicians, and radiologists. In the era of personalised and precision care, this task requires the integration, amalgamation, and combined analysis of clinical and imaging data from multiple sources. At present, multiparametric Magnetic Resonance Imaging (mpMRI) and choline–positron emission tomography (PET) are giving encouraging results; their combination allows the effective detection of local, lymph nodal, and skeletal recurrences at low PSA levels. Future diagnostic perspectives include the clinical implementation of PET/MRI scanners, multimodal fusion imaging platforms for retrospective co-registration of PET and MR images, real-time transrectal ultrasound/mpMRI fusion imaging, and novel organ-specific PET radiotracers.

## Background and discussions

Measurement of Prostate Specific Antigen (PSA) in serum is a cornerstone in the monitoring of asymptomatic PCa cancer patients after curative treatment, including both RP and RT. A rapidly increasing PSA level (i.e., high PSA velocity and short PSA doubling time) is usually related to the presence of distant metastases (i.e., skeletal or lymph nodal), while a slow and progressive increase in serum PSA concentration is often because of local disease recurrence [1].

In patients with biochemical recurrence, imaging plays a key role in the identification of the site of PCa recurrence, and imaging documentation is often required for establishing an appropriate second–line treatment [2, 3]. A palpable nodule at digital rectal examination (DRE) accompanied by a rising PSA level are signs of local disease recurrence [3]. However, DRE and transrectal ultrasound (TRUS) are neither sensitive nor specific in detecting local recurrences after RP and RT [4]. After both RP and RT, advanced multimodality imaging can be successfully employed to provide a detailed documentation of the PCa relapse, including local, lymph nodal, and skeletal recurrences, and also for establishing an appropriate second–line treatment.

### The past: TRUS, bone scintigraphy and abdominopelvic CT

#### Transrectal ultrasound (TRUS) and TRUS-guided biopsy

TRUS has a low diagnostic accuracy in the detection of local PCa recurrence after RP because of several limitations, which mainly include isoechoic lesions, post-treatment changes of the normal pelvic anatomy, and small volume recurrences. Locally recurrent PCa is hypoechoic in about 65% of cases, while about 30% of local recurrences have the same echogenicity of the vesicourethral anastomosis [5, 6]. When TRUS enables the identification of suspected areas in the prostatic fossa, the use of TRUS-guidance for targeting the biopsy procedure increases the rate of positive sampling [7]. However, the ability of TRUS to detect a local recurrence strongly depends on PSA levels. In particular, TRUS is able to detect every biopsy-proven local recurrence only with PSA levels >2.0 ng/mL [6]; at lower PSA levels the clinical use of TRUS is questionable. The sensitivity of anastomotic biopsies under TRUS guidance is variable, with values ranging from 40% to 71% for PSA levels >1 ng/mL, but it drops down to 14%–45% for PSA levels <1 ng/mL [2, 4]. Therefore, a TRUS-guided negative biopsy is not adequate to rule out the presence of a local recurrence. In accordance to the last guidelines of the European Urology Association [1], salvage RT is generally recommended on the basis of the evidence of biochemical recurrence without any histological proof of the local recurrence. After RT, diagnosing local recurrence is challenging because of radiation-induced fibrosis and shrinkage of the gland [2, 4]. In this setting, the reported values of sensitivity and specificity for TRUS are 49% and 57%, respectively [8]. TRUS-guided biopsy after RT has the main role of confirming or rule out the presence of local PCa recurrence; the information provided by needle biopsy is also important to assess the tumour spread in the gland and is essential for planning the salvage therapy (cryosurgery or prostatectomy) [9, 10]. After RT, prostate biopsy is not considered the reference standard for assessing treatment efficacy, but is an independent predictor of outcome [10].

#### Bone scintigraphy and computed tomography

**Figure 1.**
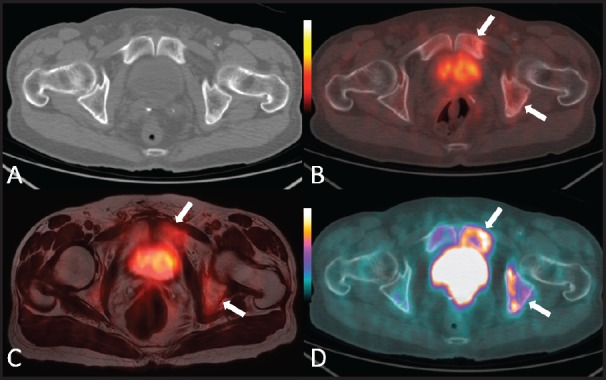
An 80-year-old man with bone metastases involving the left ischiopubic branch. The bone lesions are not detectable on the axial CT image with bone window (A) The ^18^F–choline–PET/CT (B) and the ^18^F–choline–PET/MRI fused (C) axial images show areas of moderate tracer accumulation in the left ischiopubic branch (arrows). The presence of metastatic bone involvement was further confirmed by means of ^18^F–fluoride–PET/CT scan (D) Images obtained with Quanta Prostate, Camelot Biomedical Systems s.r.l., Genoa, Italy.

Until recently, bone scintigraphy with Technetium–99m medronic acid (Tc–99m MDP) and contrast-enhanced abdominopelvic computed tomography (ceCT) have been considered the main diagnostic tools to detect skeletal and lymph nodal metastases. However, the diagnostic yield of these well-established imaging techniques is not satisfactory in patients with a serum PSA level <10 ng/mL or a low PSA velocity (<0.5 ng/mL/month) [11] (Figure 1). For example, only 12.5% of patients with biochemical failure after RP have a positive ceCT [11], and in a series of 132 men affected by biochemical failure, the mean PSA level and PSA velocity associated with a positive ceCT were 27.4 ng/mL and 1.8 ng/mL/month respectively [12]. By means of newer imaging techniques, systemic disease can be identified when PSA levels are significantly lower [2, 13].

### The present: multiparametric MRI and choline-PET

#### Multiparametric MRI

**Figure 2.**
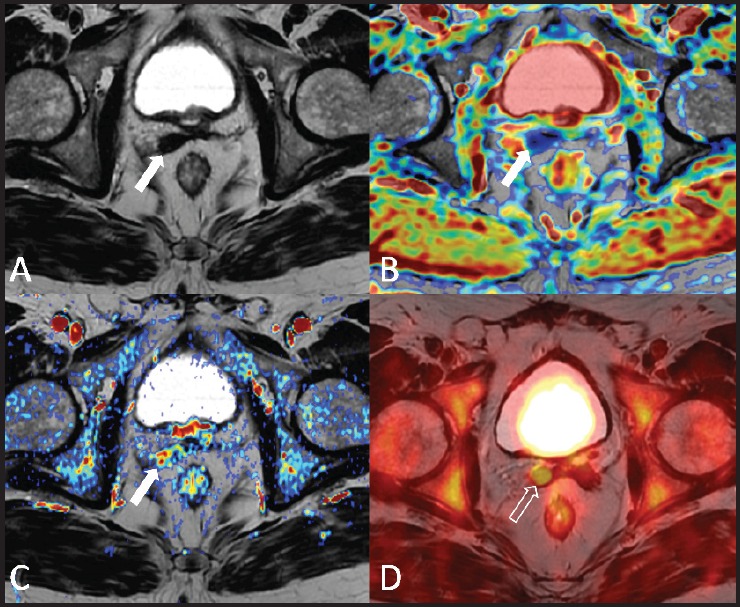
A 73-year-old patient who underwent radical prostatectomy 40 months earlier for prostate cancer Gleason 7 (3 + 4). Current PSA value of 2.2 ng/mL. The T2-weighted axial image (A) shows a hypointense nodular thickening of the remnant of the right seminal vesicle (arrow), which is characterised by restricted water diffusion on the apparent diffusion coefficient (ADC) map (B) and is hypervascular on the perfusion map obtained from the DCE–MRI acquisition (C) The ^18^F–choline–PET/MRI fused axial image (D) shows a focal accumulation of the tracer in correspondence to the remnant of the right seminal vesicle (void arrow), which confirmed the diagnosis of local relapse. Images obtained with Quanta Prostate, Camelot Biomedical Systems s.r.l., Genoa, Italy.

**Figure 3.**
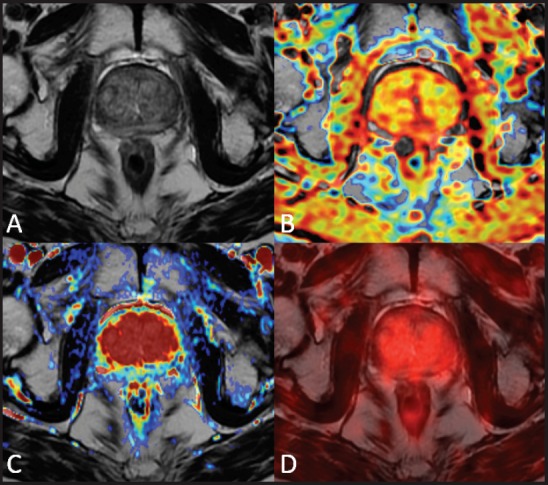
A 75-year-old patient treated with external beam radiation therapy. The T2-weighted axial image (A) shows diffuse low signal intensity of the prostate gland with loss of the normal zonal anatomy. The ADC map obtained from the diffusion-weighted sequence does not show any significant focus of restricted water diffusion (B) The transition zone is homogeneously hypervascular on the perfusion map obtained from the DCE–MRI acquisition (C) The ^18^F–choline–PET/MRI fused axial image (D) does not show any focal accumulation of the tracer in the prostate parenchyma. This case underscores that diffusely reduced signal intensity of the prostate parenchyma after RT with loss of zonal anatomy is not necessarily a sign of local recurrence. Images obtained with Quanta Prostate, Camelot Biomedical Systems s.r.l., Genoa, Italy.

Currently, the most promising diagnostic modalities for evaluating PCa patients with biochemical recurrence include mpMRI and PET/CT with radio-labeled choline derivatives. With regard to MRI, the detection of local recurrence after both RP and RT is not easy by using the information provided by morphological imaging alone (i.e., T2-weighted sequences) [14]. In mpMRI, morphological T2-weighted sequences are combined with functional techniques, including diffusion weighted imaging (DWI), dynamic contrast-enhanced (DCE) perfusion imaging, and spectroscopy [2, 3]. In the detection of local relapse after RP, T2-weighted imaging is characterised by low sensitivity (48%–61%) and specificity (52%–82%) [15, 16]; false-positive diagnoses may occur when postoperative scarring assumes a nodular appearance, mimicking recurrence. DCE–MRI is a valuable functional technique for distinguishing PCa recurrence from fibrosis in the prostatectomy fossa and remnants of normal prostatic tissue. When assessed in combination with T2-weighted imaging, DCE–MRI is particularly accurate in detecting PCa recurrence after RP with sensitivity and specificity values of 79%–88% and 89%–100% respectively [15–18] (Figure 2). After RT, radiation-induced morphological changes in the prostate include inflammation, glandular atrophy, fibrosis, and shrinkage [3, 4]. On T2-weighted images, radiation-induced changes often cause a diffusely reduced signal intensity of the gland parenchyma, more evident in the peripheral zone, with consequent loss of the physiological zonal anatomy (Figure 3). Local PCa recurrences are characterised by low-signal intensity on T2-weighted images; therefore, they are often difficult to distinguish from the surrounding irradiated prostate tissue, which has a comparable signal intensity [19, 20]. Given the low sensitivity (26%–44%) and moderate specificity (64%–86%) of T2-weighted imaging in detecting local recurrence, the use of additional functional techniques is mandatory to achieve a precise diagnosis [21–23].

#### Choline–PET/CT

Choline–PET/CT is a whole-body molecular imaging technique that is able to show the metabolic activity of phospholipid turnover within cellular membranes.

In a recent comparative study, the patient-based sensitivity, specificity, and accuracy of choline–PET/CT in diagnosing local recurrence were 54.1%, 92.3%, and 65.5% respectively, whereas those of mpMRI were 88.5%, 84.6%, and 87.4% [24]. Despite some limitations in detecting local recurrences, choline–PET/CT has the great advantage of detecting lymph-node metastases when they are not discernible at morphological imaging (i.e., ceCT and MRI) [2, 12, 13]. This peculiar feature of choline–PET/CT has an important clinical value, since up to 80% of metastatic lymph nodes in PCa have a short-axis diameter smaller than 7 mm [25], which produces a lot of false negatives at morphological imaging. Indeed, a meta–analysis by Hӧvels *et al* [26] reported a pooled sensitivity of 42% and 39% for CT and MRI respectively, and pooled specificity of 82% for both imaging techniques. Some previous studies evaluated the diagnostic yield of choline–PET/CT in lymph node staging in patients with biochemical failure after primary treatment, using lymph node dissection as standard of reference [27–29]. Scattoni *et al* reported a sensitivity of 64%, a specificity of 90%, a positive predictive value of 86%, and a negative predictive value of 72% [27]; inadequate detection of lymph node micrometastases was considered the main explanation for the low sensitivity of PET/CT. By converse, Rinnab *et al* found a 100% sensitivity for choline–PET/CT, but its positive predictive value was only 53% [28]. Another study found a high false positive rate with 30% of patients with pelvic lymph node metastases on choline–PET/CT having no pathological confirmation [29]. In accordance to the results of a meta–analysis by Umbehr *et al*, the pooled sensitivity, specificity, and the diagnostic odds ratio of choline-PET/CT were 85%, 88%, and 41.4, respectively, on a per-patient basis [30].

When considering skeletal metastases, both choline–PET/CT and mpMRI show excellent diagnostic performances [13, 14, 31]. Occasionally, choline–PET/CT may give false negatives when assessing skeletal metastases with dense sclerosis on CT; indeed choline activity tends to reduce in relation to increasing lesion sclerosis [13, 20]. By converse, sclerotic metastases are not a limitation for mpMRI by means of DWI and short tau inversion recovery (STIR) sequences [2, 3, 32]. Hence summarising, pelvic mpMRI is superior to choline–PET in depicting local relapse after RP and RT, whereas choline–PET/CT is more accurate than mpMRI in detecting lymph nodal metastatic disease; both techniques are excellent in the assessment of skeletal metastases [2, 3, 8].

### The future: multimodality fusion imaging and novel PET-radiotracers

#### Multimodality fusion imaging

**Figure 4.**
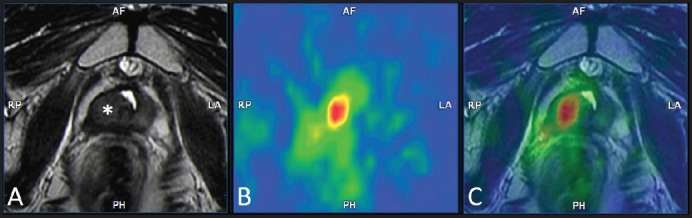
Example of bimodal ^18^F–choline–PET/MRI fusion imaging in a 78-year-old patient who had undergone external beam radiation therapy 24 months earlier for prostate cancer Gleason 7 (3 + 4). Signs of a previous TURP are also present. The T2–weighted axial image (A) shows a hypointense nodular thickening of the right lobe of the gland (asterisk). The 18F–choline–PET axial image (B) shows a focal accumulation of the tracer adjacent to the mid–line of the pelvis. The ^18^F–choline–PET/MRI fused axial image shows the precise correspondence between the MRI finding and the focal accumulation of the tracer, thus confirming the suspect of local recurrence. Images obtained with the co-registration tool of the Virtual Navigation System, Esaote Biomedica S.p.A., Genoa, Italy.

**Figure 5.**
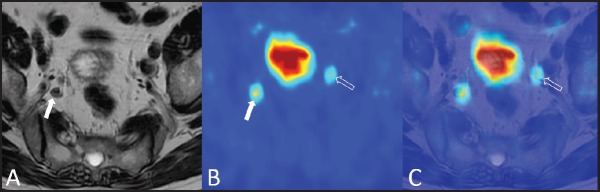
A 79-year-old man with bilateral hypogastric lymph node metastases. T2-weighted axial image (A) shows a right hypogastric lymphadenopathy (arrow). The ^18^F–choline–PET axial scan (B) demonstrates two areas of focal tracer uptake (arrow and void arrow). The ^18^F–choline–PET/MRI fused axial image (C) shows that the left focus of tracer uptake corresponded to a tiny hypogastric lymph node, which was not evident on the morphological T2-weighted image (void arrow).

**Figure 6.**
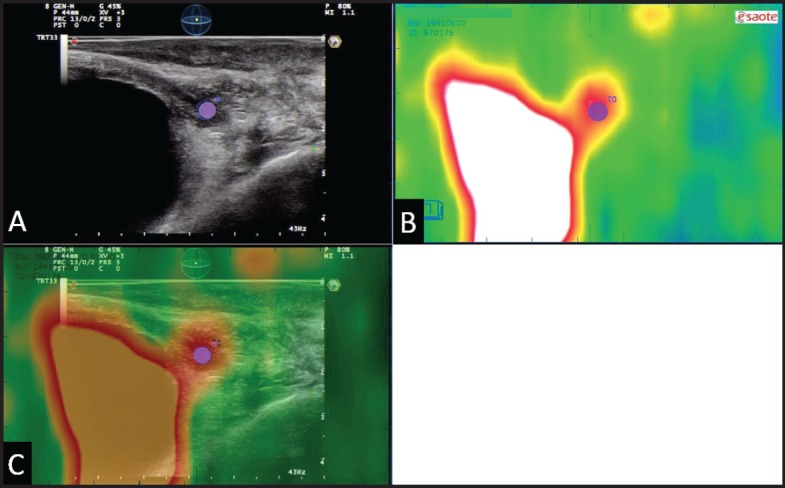
Example of ^18^F–choline–PET/TRUS fusion imaging in a 72-year-old patient treated three years earlier with radical prostatectomy for prostate cancer Gleason 8 (5 + 3). Current PSA value of 3.5 ng/mL. The longitudinal TRUS scan (A) obtained with a biplanar (linear and sectorial) endorectal probe (TRT33, Esaote, Genoa, Italy) shows an ill-defined hypoechoic area in the retrovesical space/prostatic fossa (blue marker). On the sagittal ^18^F–choline–PET image (B) the round blue marker indicates the focal tracer accumulation. The fused ^18^F–choline–PET/TRUS image (C) clearly shows the spatial correspondence between the PET–positive focus and the hypoechoic area, which was a biopsy-proven local recurrence. Images obtained with the Virtual Navigation System, Esaote Biomedica S.p.A., Genoa, Italy.

The combined and synchronised assessment of spatially aligned mpMRI and PET images by means of multimodal fusion imaging is currently giving encouraging and promising results [14]. Multimodality fusion imaging can be performed by means of simultaneous acquisition, which requires integrated PET/MRI scanners [33, 34], or retrospective co–registration of previously acquired PET and MRI datasets by means of dedicated software platforms [2, 14, 35] (Figure 4). A recent study by Piccardo *et al* has confirmed high detection rates for both modalities (i.e., 76% for 18–fluorine (18F)–choline–PET/CT and 81% for mpMRI) in patients affected by biochemical recurrence after RT [14]. Multimodality retrospective fusion imaging between choline–PET/CT and mpMRI (fused 18F–choline–PET/mpMRI) yielded an even better detection rate (i.e., 86%). These results underline that multimodal co–registration and combined interpretation are more valuable than the separate assessment of different imaging techniques [2, 14] (Figure 5). After RP and RT, the synchronous assessment of DCE–MRI and dynamic perfusion curves with the metabolic information provided by choline–PET may be beneficial to reach a correct diagnosis. Trimodal real-time fusion imaging involving TRUS, mpMRI, and choline–PET can be performed by means of dedicated software platforms implemented on high-end multipurpose ultrasound systems [2, 35, 36]. It seems to be an excellent approach to obtain histological proof of PCa recurrence after both RP and RT [2] (Figure 6). In these patients, the main salvage option is RP, but radiation-induced changes result in a higher risk of urinary incontinence and rectal injury than in the primary setting. Therefore, given the morbidity of salvage options, it is beneficial to obtain histological proof of the local recurrence before treating the patient [1, 2, 37].

#### Novel PET-radiotracers

Future perspectives in the diagnosis, follow-up, and targeted treatment of PCa also encompass the novel PET radio-tracers. Extensive studies are being conducted to develop various PET imaging agents targeting specific biomarkers of PCa, including gastrin-releasing peptide receptor (GRPR) [38] and prostate-specific membrane antigen (PSMA) [39]. GRPR-binding ligands represent potential diagnostic and therapeutic agents for the targeting of GRPR-positive tumours, including PCa. In particular, the GRPR has been shown to be overexpressed in 63%–100% of primary PCa and in more than 50% of lymph node and bone metastases [38]. The GRPR density is 26-fold higher in PCa than in prostate hyperplasia, thus providing substantial advantages over choline- and acetate-based radiotracers [40, 41]. PSMA is considered a highly specific prostate epithelial cell membrane antigen [42]. PSMA is expressed in other ‘non-target’ tissues including normal (benign) prostate epithelium, the small intestine, renal tubular cells, and salivary gland, but 100–1000 fold less than the baseline expression in PCa [43–45]. The unique functional features and the high specificity for PCa makes PSMA a theoretically perfect extracellular target for various imaging and therapeutic agents. In addition, an elevated expression of PSMA is associated with metastasis, androgen independence, and progression [46–49]. A recent study compared the diagnostic yield of ^18^F-choline–PET/CT with that of a novel PET/CT imaging technique based on a 68–Gallium (^68^Ga)–labelled PSMA ligand [50]. The detection rate was significantly higher for ^68^Ga–PSMA PET/CT, especially at low PSA levels, and all the lesions detected by choline–PET/CT were also seen on ^68^Ga–PSMA PET/CT. This result underlines that choline metabolism is not increased in a considerable number of prostate cancers, whereas the PSMA is overexpressed in most cases. Choline–PET showed lower sensitivity in detecting lymph node metastases; a significant advantage of ^68^Ga–PSMA PET/CT over choline–PET was the findings, which are typical for lymph node metastases, presented with higher contrast and excellent signal-to-background ratio [50]. In a cohort of 248 patients showing biochemical recurrence after RP, Eiber *et al* demonstrated that ^68^Ga–PSMA PET/CT is able to provide detection rates >90% at PSA levels >1 ng/mL [51]. Most importantly, the authors found a significant number of positive findings even at lower PSA values (<0.5 ng/mL). ^68^Ga–PSMA PET/CT detected lymph node metastases in 31 patients, while unenhanced CT in only one patient. Thus summarising, the main advantages of ^68^Ga–PSMA PET/CT are the sensitive detection of lesions at low PSA levels, including small lymph nodes, bone and liver metastases, primarily because of a high radiotracer uptake and low background signal [51].

Recently, promising data on PET/CT-guided salvage lymph node dissection in patients with biochemical recurrence after RP have been reported [52, 53]. Winter *et al* showed that choline–PET/CT–guided salvage resection of lymph node metastases is able to provide a long-term complete biochemical remission, without the use adjuvant therapy, in certain PCa patients with biochemical recurrence after RP [52]. In this study, histology confirmed the 13 of 16 lymph nodes suspicious on PET/CT imaging [52]. The majority of patients with histologically-confirmed lymph node metastases showed a PSA response after lymphadenectomy; in addition, three of ten patients with a single lymph node metastasis had a complete biochemical remission (PSA <0.01 ng/mL). In five patients with a single lymph node metastasis, the serum PSA decreased at values <0.02 ng/mL [52]. Therefore, in those patients showing disease relapse limited to lymph nodes, salvage lymphadenectomy seems to represent a valid therapeutic option after RP [53].

## Conclusion

The effective detection of PCa recurrence after primary treatment requires a well-trained and experienced multidisciplinary team in order to combine the morphological, functional and metabolic information coming from different techniques and imaging modalities.
